# *ACE* and *ACE2* Gene Variants Are Associated With Severe Outcomes of COVID-19 in Men

**DOI:** 10.3389/fimmu.2022.812940

**Published:** 2022-02-17

**Authors:** Laura E. Martínez-Gómez, Brígida Herrera-López, Carlos Martinez-Armenta, Silvestre Ortega-Peña, María del Carmen Camacho-Rea, Carlos Suarez-Ahedo, Paola Vázquez-Cárdenas, Gilberto Vargas-Alarcón, Gustavo Rojas-Velasco, José Manuel Fragoso, Patricia Vidal-Vázquez, Juan P. Ramírez-Hinojosa, Yunuen Rodríguez-Sánchez, David Barrón-Díaz, Mariana L. Moreno, Felipe de J. Martínez-Ruiz, Dulce M. Zayago-Angeles, Mónica Maribel Mata-Miranda, Gustavo Jesús Vázquez-Zapién, Adriana Martínez-Cuazitl, Edith Barajas-Galicia, Ludwing Bustamante-Silva, Diana Zazueta-Arroyo, José Manuel Rodríguez-Pérez, Olivia Hernández-González, Roberto Coronado-Zarco, Vania Lucas-Tenorio, Rafael Franco-Cendejas, Luis Esau López-Jácome, Rocío Carmen Vázquez-Juárez, Jonathan J. Magaña, Marlid Cruz-Ramos, Julio Granados, Susana Hernández-Doño, Diego Delgado-Saldivar, Luis Ramos-Tavera, Irma Coronado-Zarco, Gustavo Guajardo-Salinas, José Francisco Muñoz-Valle, Carlos Pineda, Gabriela Angélica Martínez-Nava, Alberto López-Reyes

**Affiliations:** ^1^ Laboratorio de Gerociencias, Dirección General, Medicina de Rehabilitación, Laboratorio de Infectología, Departamento de Reconstrucción Articular, Laboratorio de Medicina Genómica, Laboratorio Facilitador, Instituto Nacional de Rehabilitación Luis Guillermo Ibarra Ibarra, Secretaría de Salud, Mexico City, Mexico; ^2^ Postgrado en Biología Experimental, Dirección de Ciencias Biológicas y de la Salud (DCBS), Universidad Autónoma Metropolitana Iztapalapa, Mexico City, Mexico; ^3^ Departamento de Nutrición Animal, Departamento de Inmunogenética, Instituto Nacional de Ciencias Médicas y Nutrición Salvador Zubirán, Secretaría de Salud, Mexico City, Mexico; ^4^ Centro de Innovación Médica Aplicada, Subdirección de Epidemiología e Infectología, Hospital General Dr. Manuel Gea González, Mexico City, Mexico; ^5^ Departamento de Biología Molecular y Unidad de Cuidados Intensivos, Instituto Nacional de Cardiología Ignacio Chavez, Mexico City, Mexico; ^6^ Nuevo Hospital General Delegación Regional Sur de la Ciudad de México ISSSTE, Mexico City, Mexico; ^7^ Laboratorio de Biología Celular y Tisular, Laboratorio de Embriología, Escuela Militar de Medicina, Universidad del Ejército y Fuerza Aérea, Ciudad de México, Mexico; ^8^ Servicio de Cirugía General, Hospital Central Norte Petróleos Mexicanos (PEMEX), Mexico City, Mexico; ^9^ Programa de Investigadoras e investigadores por México del Consejo Nacional de Ciencia y Tecnología (CONACYT), Instituto Nacional de Cancerología, Mexico City, Mexico; ^10^ Instituto Nacional de Perinatología Isidro Espinosa de los Reyes, Mexico City, Mexico; ^11^ Independent Consultant, San Antonio, TX, United States; ^12^ Instituto de Investigación en Ciencias Biomédicas, Centro Universitario de Ciencias de la Salud, Universidad de Guadalajara, Guadalajara, Mexico

**Keywords:** COVID-19, SARS-CoV-2, *ACE2*, *ACE*, genetic variants, polymorphism

## Abstract

Severe acute respiratory syndrome coronavirus 2 (SARS-CoV-2) is responsible for the current coronavirus disease 2019 (COVID-19) pandemic, affecting more than 219 countries and causing the death of more than 5 million people worldwide. The genetic background represents a factor that predisposes the way the host responds to SARS-CoV-2 infection. In this sense, genetic variants of *ACE* and *ACE2* could explain the observed interindividual variability to COVID-19 outcomes. In order to improve the understanding of how genetic variants of *ACE* and *ACE2* are involved in the severity of COVID-19, we included a total of 481 individuals who showed clinical manifestations of COVID-19 and were diagnosed by reverse transcription PCR (RT-PCR). Genomic DNA was extracted from peripheral blood and saliva samples. *ACE* insertion/deletion polymorphism was evaluated by the high-resolution melting method; *ACE* single-nucleotide polymorphism (SNP) (rs4344) and *ACE2* SNPs (rs2285666 and rs2074192) were genotyped using TaqMan probes. We assessed the association of ACE and ACE2 polymorphisms with disease severity using logistic regression analysis adjusted by age, sex, hypertension, type 2 diabetes, and obesity. The severity of the illness in our study population was divided as 31% mild, 26% severe, and 43% critical illness; additionally, 18% of individuals died, of whom 54% were male. Our results showed in the codominant model a contribution of *ACE2* gene rs2285666 T/T genotype to critical outcome [odds ratio (OR) = 1.83; 95%CI = 1.01–3.29; p = 0.04] and to require oxygen supplementation (OR = 1.76; 95%CI = 1.01–3.04; p = 0.04), in addition to a strong association of the T allele of this variant to develop critical illness in male individuals (OR = 1.81; 95%CI = 1.10–2.98; p = 0.02). We suggest that the T allele of rs2285666 represents a risk factor for severe and critical outcomes of COVID-19, especially for men, regardless of age, hypertension, obesity, and type 2 diabetes.

## Introduction

The ongoing coronavirus disease 2019 (COVID-19) pandemic has affected around 336 million individuals, causing the death of nearly 5,560,718 severe acute respiratory syndrome coronavirus 2 (SARS-CoV-2) infected subjects around the world (1). Current evidence shows that COVID-19 patients experience clinical manifestations ranging from asymptomatic to severe pneumonia with multiple organ failure (2–4). The severity of COVID-19 and its clinical manifestations and outcomes are related to the internalization mechanism of the virus into the host cell, host genetics variants, advanced age, gender, and comorbidities (5). In this sense, the homeostasis of the renin–angiotensin system is another risk factor underlying the pathogenesis of COVID-19 because angiotensin-converting enzyme 2 (ACE2) is the predominant receptor by which the SARS-CoV-2 virus enters and infects cells (6, 7). An altered ACE/ACE2 expression ratio could contribute to severe outcomes in COVID-19 patients (8), as it does for cardiovascular diseases (9).

Recent studies have suggested the association of genetic polymorphisms of ACE and ACE2 with the case rate of COVID-19. However, the clinical implication of ACE genetic variants in the severity and prognosis of COVID-19 remains unclear. The insertion/deletion (I/D) polymorphisms play a pivotal role in cardiovascular and respiratory diseases; for instance, the D/D genotype has been associated with SARS progression (10, 11). Moreover, it has been associated with poor clinical outcomes of acute respiratory distress syndrome (ARDS), where individuals with the D/D genotype had significantly higher mortality than those who carry the I/I genotype (12). In addition, different reports have proposed that ACE2 polymorphisms rs2285666 (G870A) could modulate the susceptibility to SARS-CoV-2 infection by contributing to higher expression of ACE2 receptor (13, 14).

Furthermore, several reports have shown that ACE2 gene polymorphism is related to acute lung injury, making COVID-19 patients significantly prone to develop ARDS (12, 14, 15). To improve the understanding of how genetic variations and biological mechanisms are involved in COVID-19 severity, we consider that the association of case fatality rate and genetic variants of ACE/ACE polymorphism could represent a strategy to identify possible SNPs as susceptibility and prognostic markers in the Mexican population.

## Material and Methods

### Setting and Participants

From June 2020 to March 2021, we performed a multicenter cross-sectional study in which the following institutions were included: the Instituto Nacional de Rehabilitación Luis Guillermo Ibarra-Ibarra, Instituto Nacional de Cardiología Ignacio Chávez, Hospital Central Militar, Hospital Central Norte Pemex, and Hospital General Dr. Manuel Gea González. A non-probability sampling method was conducted, as the participants were patients at the COVID-19 triage facilities.

All subjects were evaluated with the following comprehensive clinical procedures: from each patient, oxygen saturation levels and ferritin, D-dimer, lactate dehydrogenase, and C-reactive protein (CRP) were recorded. Ferritin and D-dimer levels were measured in plasma with automatic analyzers (DxI 800 Beckman Coulter, COBAS pro e-801, COBAS integra 400 plus, and ACL TOP 550 CTS Werfem, COBAS integra 400 plus, respectively), while lactate dehydrogenase and C-reactive protein (CRP) were measured in serum using Olympus AU 800, COBAS pro c-503, COBAS integra 400 plus, and DxC 700 AU Beckman Coulter.

### Ethical Statement

This study was conducted following the good clinical practice and the Declaration of Helsinki. Informed consent was obtained from each participant before entering the study. The study was approved by the ethics committee of the Instituto Nacional de Rehabilitación LGII (INR-LGII: 17/20). The inclusion criteria were age ≥18 years and a positive test for SARS-CoV-2 infection by RT-PCR. The exclusion criteria were incomplete clinical history and related individuals.

### Outcomes

According to the criteria of Gandhi et al. (16), we defined each COVID-19 patient group as mild, severe, and critical. Mild group included those ambulatory subjects with oxygen saturation level ≥94%, and with symptoms such as fever, headache, fatigue, odynophagia, cough, rhinorrhea, diarrhea, anosmia, or dysgeusia, with or without dyspnea or pneumonia, and not requiring hospitalization. Severe status was defined as those hospitalized individuals with saturation levels <94%, and any of the following symptoms: tachypnea (FR > 30 bpm), pulmonary infiltrate >50%, and dyspnea for small efforts. Finally, the critical group were those patients requiring invasive mechanical ventilation who could course with shock and multiorgan failure. In addition, we stratified patients by their requirement of oxygen therapy in two groups: those who did not need any type of oxygen therapy and those who had oxygen requirement by nasal tips, mask, or intubation.

### Genotyping

Genomic DNA was obtained from peripheral blood and saliva using a commercial kit (QIAamp DNA blood Mini kit, Qiagen, Germany), and its quality was verified in agarose gels stained with SYBR^®^ Green I nucleic acid gel stain (Invitrogen, CA, USA). Then, the DNA concentration was measured and adjusted to 20 ng/µl with a NanoDrop spectrophotometer (Thermo Fisher Scientific, CA, USA).

A systematic review of *ACE* and *ACE2* gene SNPs was performed, from which we selected the SNPs with a minor allele frequency (MAF) ≥20% according to 1000 Genomes Project or Hapmap Project in the Mexican population (MXL) with previous reports showing significant association with other diseases.

The I/D polymorphism was genotyped by real-time polymerase chain reaction, coupled to a high-resolution melting curve (HRM) analysis allowing the identification of the dissociation temperature for the deletion and insertion amplicon. The DNA was denatured at 95°C for 5 min, followed by 40 cycles of denaturation at 95°C for 25 s, annealing at 67°C for 60 s, and extension at 72°C for 75 s, melt ramp from 77°C to 95°C. This protocol was implemented in the RotorGen Q thermal cycler (Qiagen, Germany). The genotype for this variant was assigned by HRM analysis and confirmed by the melting curve. Additionally, the rs4344 of *ACE* and rs2285666 and rs2074192 of *ACE2* were analyzed. These SNPs were determined with TaqMan genotyping assays on a StepOne Real-Time PCR equipment (Thermo Fisher Scientific, CA, USA).

### Statistical Analysis

The normality of the distribution of the variables was evaluated. Kruskal–Wallis was used for comparing non-parametric continuous variables between studied groups, and the results were described using the median and the interquartile range (IQR). For the categorical variables, the chi-squared test was performed. For all tests, a value of p <0.05 was considered statistically significant. Hardy–Weinberg expectations (HWEs) were assessed for all polymorphisms in the mild group, and the linkage disequilibrium (LD) between variants of the same gene using HaploView software V4.2 (17). A logistic regression analysis was used to evaluate the association between genetic variants and COVID-19 outcomes in the three main inheritance models, i.e., codominant, dominant, and recessive, adjusted by age, sex, hypertension, type 2 diabetes, and obesity. The final models were evaluated using the Hosmer–Lemeshow goodness-of-fit test. The association between SNPs and clinical features was assessed by comparing their distribution among alleles and genotypes by Kruskal–Wallis test and stratified by disease outcome. The analysis was performed using the STATA v.13 statistical package (StataCorp, TX, USA).

## Results

### Patients

From June 2020 to March 2021, 489 individuals with COVID-19 were selected. Nonetheless, eight patients did not have complete clinical data. Therefore, a total of 481 patients were included in this study. We classified the study population as 31% (149) mild, 26% (125) severe, and 43% (207) critical, of which 86 subjects died. The median age was 51 (IQR = 43–63), and a total of 60% (290) were male subjects. In the overall population, the common symptoms were cough, headache, myalgia, and dyspnea. Most individuals had at least one coexisting illness such as hypertension and type 2 diabetes. We observed significant differences in the majority of the subjects’ clinical characteristics depending on the group they belong to (**Table 1**). However, no significant differences were observed for abdominal pain (p > 0.05).

**Table 1:** Clinics characteristics of population of study.

	Total	Mild	Severe	Critical	*p*
	n = 481	n = 149	n = 125	n = 207	
Age (years)	51 (43,63)	44 (32,51)	53 (44,65)	58 (49,68)	<0.001*
Sex Male	290 (60%)	71 (48%)	79 (63%)	140 (68%)	0.001**
Fever	154 (32%)	49 (33%)	21 (17%)	84 (41%)	<0.001**
Cough	322 (67%)	92 (62%)	73 (58%)	157 (76%)	0.001**
Dyspnea	255 (53%)	38 (26%)	72 (58%)	145 (70%)	<0.001**
Chest pain	210 (44%)	67 (45%)	75 (60%)	68 (33%)	<0.001**
Headache	304 (63%)	121 (81%)	66 (53%)	117 (56%)	<0.001**
Odynophagia	247 (51%)	99 (66%)	63 (50%)	85 (41%)	<0.001**
Rhinorrhea	193 (40%)	87 (58%)	59 (47%)	47 (23%)	<0.001**
Myalgia	321 (67%)	104 (70%)	94 (75%)	123 (59%)	0.008**
Diarrhea	177 (37%)	64 (43%)	59 (47%)	54 (26%)	0.001**
Vomiting	80 (17%)	34 (23%)	23 (18%)	23 (11%)	0.01**
Abdominal pain	46 (10%)	19 (13%)	7 (6%)	20 (10%)	0.13**
Anosmia	245 (51%)	65 (44%)	100 (80%)	80 (39%)	<0.001**
Dysgeusia	261 (54%)	82 (55%)	97 (78%)	82 (40%)	<0.001**
Obesity	129 (27%)	16 (11%)	35 (28%)	78 (38%)	0.001**
Overweight	152 (32%)	18 (12%)	46 (37%)	88 (42%)	<0.001**
Type 2 Diabetes	134 (28%)	14 (9%)	44 (35%)	76 (37%)	<0.001**
Hypertension	112 (23%)	12 (8%)	37 (30%)	63 (30%)	<0.001**

Text in bold denotes statistical significance.

*Kruskal–Wallis test.

**Chi-squared test.

### Association Between *ACE* and *ACE2* Polymorphisms With COVID-19 Outcomes

Allelic and genotype frequencies were calculated (**Table 2**). For *ACE* gene, I/D alleles in the total sample were 61% for I and 39% for D allele; in addition, for rs4344 polymorphism, the MAF was 39% (A allele). Only 42% of patients with mild illness carried the II genotype, while 33% of severe patients and 39% of critical patients showed this genotype. These frequencies were also observed for the rs4344 variant and were not significantly different between study groups.

**Table 2:** Allelic and genotypes frequencies of the population of study.

	Frequencies (%)				p*	Frequencies (%)		p**
	Total	Mild	Severe (n	Critical		HWE	Oxygen requirement (n = 181)	
	(n = 481)	(n = 149)	= 125)	(n = 207)				
ACE gene polymorphisms								
I/D								
I	587 (61%)	188 (63%)	147 (59%)	252 (61%)	0.58		399 (60%)	0.38
D	375 (39%)	110 (37%)	103 (41%)	162 (39%)			265 (40%)	
II	183(38%)	62(42%)	41(33%)	80(39%)	0.56	0.37	121 (37%)	0.55
ID	221(46%)	64(43%)	65(52%)	92(44%)			157 (47%)	
DD	77(16%)	23(15%)	19(15%)	35(17%)			54 (16%)	
rs4344								
G	590 (61%)	192 (64%)	149 (60%)	250 (60%)	0.43		399 (60%)	0.54
A	372 (39%)	106 (36%)	101 (40%)	163 (39%)			264 (40%)	
GG	183(38%)	63(42%)	41(33%)	79(38%)	0.33	0.59	120 (36%)	0.73
GA	224(47%)	66(44%)	67(54%)	91(44%)			158 (48%)	
AA	73(15%)	20(13%)	17(14%)	36(17%)			53 (16%)	
ACE2 gene polymorphisms								
rs2285666								
C	567 (59%)	190 (64%)	146 (58%)	231 (56%)	0.10		377 (57%)	0.05
T	395 (41%)	108 (36%)	104 (42%)	183 (44%)			287 (43%)	
CC	241 (50%)	76(51%)	63 (50%)	102(49%)	**0.009**	**<0.001**	165 (50%)	**0.002**
CT	85 (18%)	38(25%)	20 (16%)	27 (13%)			47 (14%)	
TT	155 (32%)	35(23%)	42 (34%)	78 (38%)			120 (36%)	
rs2074192								
C	556 (58%)	177 (59%)	137 (55%)	242 (58%)	0.52		379 (57%)	0.63
T	406 (42%)	121 (41%)	113 (45%)	172 (42%)			285 (43%)	
CC	233 (48%)	69 (46%)	58 (46%)	106 (51%)	**0.05**	**<0.001**	164 (49%)	**0.01**
CT	90 (19%)	39 (26%)	21 (17%)	30 (14%)			51 (15%)	
TT	158 (33%)	41 (28%)	46 (37%)	71 (34%)			117 (35%)	

Chi-square test. Text in bold denotes statistical significance.

*Mild vs. severe and critical.

**Mild vs. oxygen requirement.

HWE, Hardy–Weinberg equilibrium.

When we compared the genotypes frequencies of the evaluated SNPs between mild, severe, and critical groups, we found significant differences in rs2285666 polymorphism and marginally difference in rs2074192 polymorphism both of *ACE2* gene (*p* = 0.009, *p* = 0.05, respectively). When comparing mild illness and the requirement for oxygen therapy (severe or critical illness), we found significant differences in rs2285666 and rs2074192 *ACE2* variants genotypic frequencies (*p* = 0.002 and *p* = 0.01, respectively). Among those patients that needed oxygen therapy, 36% carried the T/T genotype of the rs2285666 gene variants in comparison to 23% of the patients who did not need it, while for the variant of the rs2074192, the T/T genotype showed a 35% in comparison to 28% of the patients who did not require it. For the alleles frequencies, the MAF of rs2285666 variant was 41% (T allele), while the MAF of rs2071192 was 42% (T allele). We did not detect any significant difference in the allelic frequencies of both polymorphisms between outcomes.

The genotypes of *ACE* I/D and rs4344 variants were in Hardy–Weinberg equilibrium. However, the genotypes of *ACE2* were not in equilibrium. We observed a strong LD between both *ACE* gene variants, showing a D′ of 0.97. Regarding the *ACE2* gene variants, the LD was slightly lower, having a D′ of 0.88.

### Multiple Logistic Regression Analysis

In the logistic regression analysis, we did not find a statistically significant association of *ACE* variants with COVID-19 outcomes. Notwithstanding, for the C/T genotype of the rs2285666 polymorphism, we found an OR = 0.52 (95%CI = 0.29–0.94), and for T/T genotype, an OR = 1.66 (95%CI = 1.01–2.73) in the codominant model with critical outcome (**Supplementary Material**). After adjusting by age, hypertension, sex, type 2 diabetes, and obesity, we found a significant positive association between the T/T genotype in the codominant model of rs2285666 polymorphism and critical outcome (OR = 1.83; 95%CI = 1.01–3.29), and for oxygen requirement (OR = 1.76; 95%CI = 1.01–3.04). Moreover, for this same *ACE2* genetic variant but under a recessive model, we observed an OR = 1.89 (95%CI = 1.06–3.35) for critical outcome and an OR = 1.80 (95%CI = 1.06–3.05) for oxygen requirement. This positive association with critical outcome and oxygen requirement found with the T/T genotype of *ACE2* rs2285666 polymorphism was also observed with the T allele (OR = 1.58, 95%CI = 1.09–2.30; and OR = 1.52, 95%CI = 1.08–2.14). Finally, the T allele of this *ACE2* variant was marginally associated with a higher risk of severe outcome (OR = 1.45; 95%CI = 0.99–2.13) (**Table 3**).

**Table 3:** Association of ACE and ACE2 polymorphism with the outcome of COVID-19.

Polymorphisms	Severe			Critical			Oxygen requirement		
	OR*	95%CI	*p*	OR*	95%CI	*p*	OR*	95%CI	*p*
ACE I/D									
Codominant									
II	Reference			Reference			Reference		
ID	1.21	0.67-2.15	0.52	0.82	0.47-1.44	0.50	0.98	0.59-1.64	0.95
DD	1.17	0.53-2.57	0.69	1.09	0.52-2.3	0.81	1.12	0.57-2.22	0.72
Dominant									
ID+DD^d^	1.20	0.69-2.07	0.50	0.89	0.53-1.50	0.67	1.02	0.64-1.64	0.92
Recessive									
DD^r^	1.05	0.51-2.06	0.89	1.21	0.61-2.40	0.57	1.13	0.61-2.13	0.68
Alleles									
I	Reference			Reference			Reference		
D	1.11	0.75-1.62	0.59	1.00	0.69-1.44	0.99	1.05	0.75-1.47	0.77
ACE rs4344									
Codominant									
GG	Reference			Reference			Reference		
GA	1.31	0.73-2.33	0.35	0.90	0.51-1.56	0.71	1.07	0.64-1.78	0.78
AA	1.17	0.51-2.66	0.70	1.28	0.60-2.74	0.51	1.23	0.62-2.49	0.54
Dominant									
GA+AA^d^	1.29	0.74-2.22	0.36	0.99	0.58-1.66	0.97	1.11	0.69-1.78	0.65
Recessive									
AA^r^	1.00	0.47-2.13	0.98	1.36	0.67-2.74	0.38	1.19	0.62-2.28	0.59
Alleles									
G	Reference			Reference			Reference		
A	1.13	0.77-1.66	0.52	1.08	0.75-1.57	0.52	1.11	0.79-1.55	0.55
ACE2 rs2285666									
Codominant									
CC	Reference			Reference			Reference		
CT	1.02	0.44-2.37	0.95	0.99	0.43-2.26	0.99	0.88	0.42-1.87	0.75
TT	1.64	0.89-3.01	0.11	**1.83**	**1.01-3.29**	**0.04**	**1.76**	**1.01-3.04**	**0.04**
Dominant									
CT+TT^d^	1.37	0.79-2.39	0.26	1.49	0.87-2.54	0.140	1.44	0.88-2.35	0.14
Recessive									
TT^r^	1.69	0.93-3.06	0.08	**1.89**	**1.06-3.35**	**0.03**	**1.80**	**1.06-3.05**	**0.03**
Alleles									
C	Reference			Reference			Reference		
T	1.45	0.99-2.13	0.05	**1.58**	**1.09-2.30**	**0.01**	**1.52**	**1.08-2.14**	**0.01**
ACE2 rs2074192									
Codominant									
CC	Reference			Reference			Reference		
CT	0.91	0.39-2.09	0.82	0.86	0.38-1.96	0.73	0.88	0.43-1.83	0.74
TT	1.23	0.68-2.24	0.49	1.09	0.61-1.94	0.76	1.15	0.67-1.95	0.61
Dominant									
CT+TT^d^	1.12	0.66-1.92	0.66	1.01	0.60-1.70	0.95	1.06	0.66-1.69	0.81
Recessive									
TT^r^	1.25	0.70-2.25	0.44	1.12	0.63-1.96	0.69	1.17	0.69-1.96	0.55
Alleles									
C	Reference			Reference			Reference		
T	1.16	0.80-1.69	0.43	1.05	0.73-1.51	0.3	1.09	0.78-1.53	0.57

Text in bold denotes statistical significance.

*Adjusted for age, sex, hypertension, type 2 diabetes, and obesity.

d, dominant inheritance model, the reference group is formed by mayor allele homozygote genotype; r, recessive inheritance model, the reference group is formed by mayor allele homozygote and heterozygote genotype.

Given that *ACE2* gene polymorphisms are in the X chromosome, an allele analysis stratified by sex was performed. We observed that the positive association found with the rs2285666 T allele and severe illness was maintained and even had a higher magnitude and significance among men than the one observed in the whole study population (OR = 1.72; 95%CI = 1.02–2.89; *p* =0.03). The same happened with the association seen between *ACE2* rs228566 T allele and critical illness, and with oxygen requirement, which for men showed an OR = 1.81 (95%CI = 1.10–2.98) and OR = 1.77 (95%CI, 1.12–2.83), respectively (**Table 4**).

**Table 4:** Association of ACE2 alleles with COVID-19 outcome in men.

Gen	Severe			Critical			Oxygen requirement		
	OR*	95%CI	*p*	OR*	95%CI	*p*	OR*	95%CI	*p*
**ACE2 rs2285666**									
**C**	–			–					
**T**	**1.72**	**1.02-2.89**	**0.03**	**1.81**	**1.10-2.98**	**0.02**	**1.77**	**1.12-2.83**	**0.01**
**ACE2 rs2074192**									
**C**	–			–					
**T**	1.22	0.75-2.00	0.41	0.97	0.61-1.56	0.92	1.07	0.69-1.66	0.75

Text in bold denotes statistical significance.

*Adjusted for age, hypertension, type 2 diabetes and obesity.

**Figure 1:**
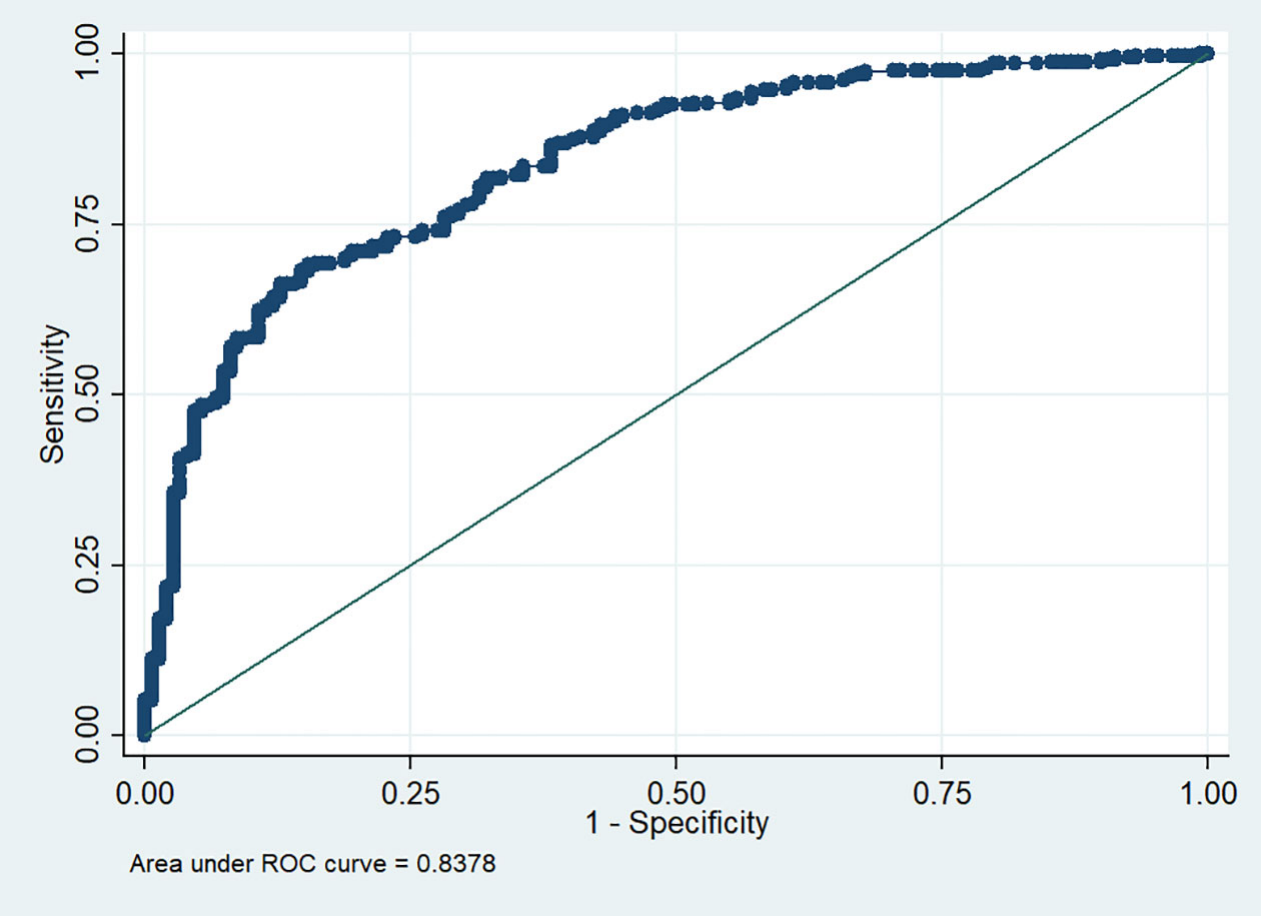
ROC curve for the association model of rs2285666 ACE2 polymorphism with oxygen requirement.

We further evaluated the potential to identify patients who needed oxygen therapy using the predicted values of the multivariate logistic regression model of the associated polymorphism of *ACE2* by receiver operating characteristic (ROC) curve analysis. We observed that with this model, we can categorize patients that required oxygen therapy from those who did not (**Figure 1**).

### Association of *ACE* and *ACE2* Genes Polymorphisms With COVID-19 Clinical Biomarkers and Oxygen Saturation Levels

We performed a refined approach to determine the behavior of the main COVID-19 clinical biomarkers and oxygen saturation level in our study population. In this sense, we could observe a significant reduction in oxygen saturation with increasing disease severity. Furthermore, statistically significant differences were observed among outcomes for COVID-19 biomarkers (*p* < 0.001) (**Supplementary Table S2**). D-Dimer and ferritin plasma concentrations showed significantly higher values in critical COVID-19 subjects (679.5 ng/ml for D-dimer and 619 ng/ml for ferritin) than in mild disease patients. We also evaluated lactate dehydrogenase and CRP in serum. A clear tendency for increased levels of these biomarkers was observed among outcomes.

We evaluated the distribution of clinical features and biomarkers among the four genotypes polymorphisms and COVID-19 outcomes; nevertheless, we only found significant differences with the *ACE2* rs2285666 polymorphism. In the mild group, we found a higher amount of D-dimer in T allele carriers (p50 = 323 ng/ml, IQR = 217–501 ng/ml) in comparison to C allele carriers, and for C/T genotype (p50 = 360 ng/ml, IQR = 214.5–501 ng/ml) and T/T genotype (p50 = 285, IQR = 217,430 ng/ml) carriers in comparison to C/C patients. Regarding ferritin levels, differences among genotypes were also observed among mild, severe, and critical patients. In this context, the lowest level of ferritin was observed in the carriers of the heterozygote genotype. Moreover, CPR was significantly increased in T allele carriers of the mild group (p50 = 4.95 mg/ml, IQR = 2–13.4 mg/ml), and in the critical patients with the T/T genotype (p50 = 26.55 mg/ml, IQR = 16.61–113.19 mg/ml). In the severe group, we only found significant differences in lactate dehydrogenase levels among *ACE2* rs2285666 genotypes, showing the highest level those patients carrying the T/T genotype (p50 = 322.8 U/L, IQR = 213.3–439 U/L) (**Supplementary Table S3**).

## Discussion

A total of 481 individuals with COVID-19 were included in this study, 22% of them were in a severe stage and 86 subjects died with a median age of 64.5 years. These findings agree with those reported by Zhang et al., since he observed that patients older than 60 years were more prone to develop complications with fatal outcomes (18). Additionally, COVID-19 has been reported to be more severe in male individuals (19), which is in agreement with our results. The 67% of individuals who developed a critical outcome were male, and 28 (67%) of the total deaths were attributable to them. This difference in gender susceptibility has been reported in previous studies performed in Italy (20), Spain, China, Germany, the UK, and South Korea (21).

A recent meta-analysis showed that fever, cough, fatigue, and dyspnea were the most prevalent symptoms of COVID-19 (22). On the other hand, in the present work, the majority of symptoms were significantly different among the outcomes (*p* < 0.001); cough and dyspnea were the highlights in critical disease conditions. A previous report from the WHO–China Joint Mission on COVID-19 showed that 87.9% of confirmed COVID-19 cases presented fever, while in our study, only 32% had fever. Interestingly, 33% of mild patients in our study presented fever, while 41% of those who developed a critical illness had fever. Cough is another common symptom with a previously reported prevalence of 67.7% (4). In our study, we observed a 67% of frequency; however, in critical cases, it was major than previously reported (76%).

Frequent comorbidities observed in individuals with COVID-19 include hypertension, type 2 diabetes, and cardiovascular disease, being serious risk factors in severely ill individuals compared with non-severely ill patients (23). In our study, critically ill adults with COVID-19 presented type 2 diabetes (28%), obesity (27%), and hypertension (23%). These findings are consistent with the published data by Wu et al. (24) and Grasselli et al. (25).

Some risk factors have been described for COVID-19 outcomes; however, understanding the role of host genetic variants for risk or protective effect could provide some insights into COVID-19 outcomes. Differences in the severity of COVID-19 due to sex have already been reported where men are the most vulnerable to severe or fatal COVID-19 outcomes (26, 27). Possible explanations include exposure to environmental factors such as smoking, diet, and physical activity, and genetic factors associated with the mechanisms of infection by SARS-CoV-2 including the genes of the *ACE/ACE2* pathways.

Although there are several studies stating the association of different polymorphisms of *ACE* and *ACE2* with hypertension and other cardiovascular diseases, there is no consistent evidence describing the role of rs2285666 and rs2074192 of *ACE2* gene or of I/D and rs4344 polymorphisms of *ACE g*ene in COVID-19 outcomes.

Regarding *ACE* I/D polymorphisms, the frequencies observed in our study were similar to those reported in the Mexican Mestizo population by Vargas-Alarcón et al. They indicated that the I/D polymorphism distinguishes the Amerindian population from other populations, suggesting that the I/D polymorphism could be a distinctive genetic susceptibility marker for some diseases in Amerindian population (28). Sarangarajan et al. reported the prevalence of I/D in different populations including South Americans with I/D prevalence similar to our findings; it is important to state that I/D genotypes prevalence were different among geographical locations (29). This evidence points out the consistency of our results regarding *ACE* I/D variant frequencies.

Some studies of *ACE g*ene in the Mexican population have reported the I/D polymorphism as a genetic marker of susceptibility for cardiovascular disease, diabetes, and hypertension diseases (28, 30). This evidence suggests that the I/D polymorphism of *ACE* could impact COVID-19 outcomes (31, 32). A recent study by Gómez et al. associated the D/D genotype of *ACE* only with hypertensive individuals who presented a severe COVID-19 outcome (12). Conversely, our results showed no significant association of this variant with COVID-19 outcomes, but we found a significant association with hypertension that could be an indirect effect of COVID-19 complications (see **Supplementary Material**). In agreement with our results, Karakas et al. reported that I/D was not associated with the severity of COVID-19 infection in Turkey populations (33). Nevertheless, it is important to point out that the D/D genotype of *ACE* gene has a role on the renin–angiotensin system, by increasing ACE levels and angiotensin-inactivating AT-1 receptor, and downstream pathophysiological effects. Therefore, future studies should not be discarded. In the same way, rs4344 polymorphism has been associated with hypertension (34) and angiotensin-converting enzyme inhibitors-related cough (15). Here, we observed the same results; nonetheless, no association with critical COVID-19 outcomes was identified.

The *ACE2* rs2285666 variant falls in the third intron of the gene, affecting gene expression by an alternative splicing mechanism (7). Asselta and colleagues reported that substitution of C for T increased the strength of the splice site in 9.2%, having as result a higher expression of the ACE2 protein (35). Gemmati and Tisato reported that the *ACE2* gene is a “first genetic gateway” involved in infection, severity, and COVID-19 (26); however, differences among populations and geographic regions could exist, as reported by Shikov et al. (36). Moreover, *ACE2* has been associated with hypertension, heart failure, and diabetes. Falahi and Kenarkoohi have already suggested that more studies focused on the role of *ACE2* gene in the pathogenesis and outcomes of COVID-19 are needed due to the existing controversies whether *ACE2* genotypes might explain the differences in the infection severity (37). Our results showed that the T allele of rs2285666 is a risk factor of severe and critical outcomes in COVID-19, especially in men. Choudhary et al. described the possible physiological roles of *ACE2* in COVID-19, suggesting that the study of *ACE2* variants will help understand the pathophysiology of this disease (14). Moreover, Benetti et al. reported in a cohort of COVID-19 patients that genetic variants of *ACE2* have an impact on its protein function, which reinforces the hypothesis that at least some variants identified, or the cumulative effect of them, might confer different susceptibility to infection and progression of COVID-19 (38). Our results indicate an association between the T/T genotype of *ACE2* rs2285666 (G8790A) and an increased risk for severe–critical outcomes (oxygen requirement) in individuals with COVID-19. Srivastava and colleagues stated that genetic variations of *ACE2* affect the susceptibility to COVID-19 and found a lower infection rate in the carriers of the T allele in Indian populations. However, it is important to mention that there are many factors involved in the SARS-CoV2 infection, and *ACE2* might not be the only gene involved (39). For the rs2074192 *ACE2* polymorphism, Shikov et al. (36) reported similar frequencies between controls, mild, and severe outcome of COVID-19 with the ones presented here. However, a recent study by Cafiero et al. (40) reported in 104 subjects of Italy that even though genotypes frequencies were similar to our results (frequencies CC>TT>CT), they suggested that rs2074192 could predict the clinical outcome of COVID-19. We did not find a significant association between this *ACE2* variant and disease outcome; nevertheless, this could be explained by the difference in sample sizes and the difference in the genetic background among both populations.

Regarding risk differences by sex, Gemmati et al. reported that an unbalance of *ACE/ACE2* might show marked differences in the outcomes of COVID-19 in both sexes, given that this unbalance could induce a higher inflammatory mediators/receptors expression; hence, men might show a worse clinical scenario than women, as women could activate a mosaic advantage due to their X-heterozygosity (27). After all, many genes associated with the regulation of the immune system are located in the X chromosome. In that sense, *ACE2* gene locus is Xp22.2, and it has been reported that the rs2285666 T/T genotype of *ACE2* decreased the gene expression level by up to 50% compared with the C/C genotype in Italian and other European populations (27, 38). Supporting this notion, we found that men had a higher risk of severe and critical COVID-19 disease than women carrying the T allele of this *ACE2* polymorphism.

Among the clinical features evaluated in the present study that have been used as biomarkers for COVID-19, we could observe that *ACE2* genetic variant had an impact on D-dimer, ferritin, lactate dehydrogenase, and CRP levels. Even though the direct effect of *ACE2* on these biomarkers levels is not quite clear, the difference showed in these COVID-19-associated biomarkers supports the notion that *ACE2* rs228566 polymorphism could be consider as a genetic susceptibility marker for COVID-19 outcome.

Another factor important to mention is that there are no previous studies of *ACE2* genetic variants in the Mexican population with COVID-19, although the frequency difference of these variants among different populations could affect the association of *ACE2* with COVID-19 illness. However, not only the genetic background might explain the differences in the outcomes of COVID-19, but also environmental risk factors such as smoking, drinking habits, and personal hygiene could be implicated.

The strengths of this study include a representative and multicentric hospital-based from Mexico City sample, obtaining clinical information, and the diagnosis of COVID-19 by RT-PCR test and blood sample for DNA extraction. Additionally, the patient outcomes were classified by Gandhi criteria. This adequate classification allowed us to stratify patients and to identify the association of genetic variants with the outcomes of individuals with COVID-19. However, the limitations of this study include that we could not have lifestyle information such as smoking habits, which has been reported to increase the severity of COVID-19 outcomes in men. Another limitation worth mentioning is that we could not evaluate other genes involved in ACE and ACE2 pathways that could be contributing to the increased risk for severe COVID-19 outcome seen for the T allele of *ACE2* rs228566 polymorphism.

In conclusion, our results show that the T allele of rs2285666 is associated with more severe outcomes of COVID-19, particularly in men, regardless of their age or the presence of hypertension and type 2 diabetes. This genetic variant could be useful as a prediction and susceptibility marker in the prognosis of COVID-19, which will also help to personalize the COVID-19 treatment.

## Data Availability Statement

The original contributions presented in the study are included in the article/**Supplementary Material**. Further inquiries can be directed to the corresponding authors.

## Ethics Statement

This study was conducted following the good clinical practice and the Declaration of Helsinki. Informed consent was obtained from each participant before entering the study. The study was approved by the ethics committee of (INR-LGII: 17/20). The patients/participants provided their written informed consent to participate in this study.

## Author Contributions

ALR, GAMN, and CP: study conception and design. LEMG, CSA, GGS, GVZ, and PVC: literature search. RFC, LLJ, RVJ, JRH, RVV, YRS, RBD, FMR, MMM, MCR, DZA, EBG, LBS, DZA, and ICZ: included patients. LEMG, SHD, DDS, LRT, AMC, and VLT: acquisition of data. LEMG, CCR, OHG, JMRP, SOP, BHL, JMF, and GRV: performed experiments. LMG, GMN, and JJM: data analysis and interpretation. CMA: figures. LEMG, CMA, and ALR: drafting the article. GVA, GJ, FMV, GMN, and ALR: critical revision of the article and final approval of the version to be published. All authors contributed to the article and approved the submitted version.

## Funding

This study was funded by the Consejo Nacional de Ciencia y Tecnología; CONACYT 312513 SARS-COV 2.

## Conflict of Interest

The authors declare that the research was conducted in the absence of any commercial or financial relationships that could be construed as a potential conflict of interest.

## Publisher’s Note

All claims expressed in this article are solely those of the authors and do not necessarily represent those of their affiliated organizations, or those of the publisher, the editors and the reviewers. Any product that may be evaluated in this article, or claim that may be made by its manufacturer, is not guaranteed or endorsed by the publisher.
