# Supplementary material for: ACE and ACE2 Gene Variants Are Associated With Severe Outcomes of COVID-19 in Men
Source: Front Immunol. 2022 Feb 17;13:812940. doi: 10.3389/fimmu.2022.812940 (PMC8892378; doi:10.3389/fimmu.2022.812940)
Supplement: Supplementary file 1 [file DataSheet_1.docx]

Table 1S.- Association of ACE and ACE2 polymorphism with outcome of COVID-19

| **Polymorphisms** | **Severe** | | | **Critical** | | | **Oxygen requirement** | | |
| --- | --- | --- | --- | --- | --- | --- | --- | --- | --- |
|  | **OR*** | **IC** | **P value** | **OR*** | **IC** | **P value** | **OR*** | **IC** | **P value** |
| **ACE I/D** | | | | | | | | | |
| Codominant |  |  |  |  |  |  |  |  |  |
| II | Reference |  |  | Reference |  |  | Reference |  |  |
| ID | 1.53 | 0.90-2.59 | 0.11 | 1.11 | 0.70-1.76 | 0.64 | 1.25 | 0.82-1.91 | 0.29 |
| DD | 1.24 | 0.60-2.57 | 0.54 | 1.17 | 0.63-2.19 | 0.60 | 1.20 | 0.67-2.14 | 0.53 |
| Dominant |  |  |  |  |  |  |  |  |  |
| ID+DD^d^ | 1.46 | 0.88-2.39 | 0.13 | 1.13 | 0.73-1.73 | 0.57 | 1.24 | 0.83-1.84 | 0.28 |
| Recessive |  |  |  |  |  |  |  |  |  |
| DD^r^ | 1.5 | 0.91-2.46 | 0.46 | 1.18 | 0.76-1.18 | 0.46 | 1.06 | 0.62-1.81 | 0.82 |
| Alleles |  |  |  |  |  |  |  |  |  |
| I | Reference |  |  | Reference |  |  | Reference |  |  |
| D | 1.19 | 0.88-1.81 | 0.19 | 1.09 | 0.51-1.19 | 0.26 | 1.13 | 0.85-1.50 | 0.38 |
| Rs4344 | | | | | | | | | |
| Codominant |  |  |  |  |  |  |  |  |  |
| GG | Reference |  |  | Reference |  |  | Reference |  |  |
| GA | 1.55 | 0.92-2.62 | 0.09 | 1.09 | 0.69-1.73 | 0.68 | 1.25 | 0.82-1.91 | 0.29 |
| AA | 1.30 | 0.61-2.78 | 0.49 | 1.43 | 0.75-2.71 | 0.27 | 1.39 | 0.76-2.52 | 0.28 |
| Dominant |  |  |  |  |  |  |  |  |  |
| GA+AA^d^ | 1.50 | 0.91-2.46 | 0.11 | 1.17 | 0.76-0.96 | 0.74 | 1.28 | 0.86-1.91 | 0.21 |
| Recessive |  |  |  |  |  |  |  |  |  |
| AA^r^ | 0.96 | 0.46-1.99 | 0.91 | 1.14 | 0.51-2.55 | 0.74 | 1.22 | 0.70-2.14 | 0.46 |
| Alleles |  |  |  |  |  |  |  |  |  |
| G | Reference |  |  | Reference |  |  | Reference |  |  |
| A | 1.22 | 0.86-1.73 | 0.25 | 1.18 | 0.87-1.61 | 0.28 | 1.20 | 0.90-1.59 | 0.21 |
| Rs2285666 | | | | | | | | | |
| Codominant |  |  |  |  |  |  |  |  |  |
| CC | Reference |  |  | Reference |  |  | Reference |  |  |
| CT | 0.63 | 0.33-1.19 | 0.16 | **0.52** | **0.29-0.94** | **0.03** | **0.56** | **0.34-0.94** | **0.03** |
| TT | 1.44 | 0.82-2.53 | 0.19 | **1.66** | **1.00-2.7** | **0.04** | 1.57 | 0.99-2.51 | 0.05 |
| Dominant |  |  |  |  |  |  |  |  |  |
| CT+TT^d^ | 1.02 | 0.63-1.64 | 0.92 | 1.07 | 0.70-1.63 | 0.46 | **1.33** | **1.01-1.77** | **0.04** |
| Recesive |  |  |  |  |  |  |  |  |  |
| TT^r^ | 1.63 | 0.93-2.82 | 0.08 | **2.02** | **1.10-3.71** | **0.02** | **1.78** | **1.09-2.89** | **0.02** |
| Alleles |  |  |  |  |  |  |  |  |  |
| C | Reference |  |  | Reference |  |  | Reference |  |  |
| T | 1.25 | 0.89-1.76 | 0.20 | 1.39 | 1.02-1.89 | **0.03** | **1.84** | **1.18-2.86** | **0.006** |
| **ACE2 rs2074192** | | | | | | | | | |
| Codominant |  |  |  |  |  |  |  |  |  |
| CC | Reference |  |  | Reference |  |  | Reference |  |  |
| CT | 0.64 | 0.33-1.20 | 0.17 | 0.50 | 0.28-0.88 | **0.02** | 0.55 | 0.33-0.90 | **0.02** |
| TT | 1.33 | 0.77-2.30 | 0.30 | 1.33 | 0.69-1.83 | 0.63 | 1.20 | 0.76-1.88 | 0.42 |
| Dominant |  |  |  |  |  |  |  |  |  |
| CT+TT^d^ | 0.99 | 0.61-1.60 | 0.98 | 0.82 | 0.53-1.25 | 0.36 | 0.88 | 0.59-1.390 | 0.53 |
| Recessive |  |  |  |  |  |  |  |  |  |
| TT^r^ | 1.39 | 0.82-2.36 | 0.22 | 1.28 | 0.70-2.33 | 0.41 | 1.43 | 0.93-2.19 | 0.09 |
| Alleles |  |  |  |  |  |  |  |  |  |
| C | Reference |  |  | Reference |  |  | Reference |  |  |
| T | 1.20 | 0.85-1.69 | 0.27 | 1.03 | 0.76-1.40 | 0.80 | 1.10 | 0.83-1.45 | 0.50 |

*d: dominant inheritance model, the reference group is formed by mayor allele homozygote genotype; r: recessive inheritance model, the reference group is formed by mayor allele homozygote and heterozygote genotype. Text in bold denotes statistical significance

Table 2S.- Clinics and biomarkers characteristics of population of study

|  | Total  n=446 | Mild  n=132 (45%) | Severe n=115 (33%) | Critical  n=199 (22%) | P* |
| --- | --- | --- | --- | --- | --- |
| Oxygen saturation % | 88(80,93) | 94(92,95.5) | 87(75,92) | 83(72,89) | **<0.001** |
| D-dimer (ng/mL) | 410.5(202,824) | 276(171,470) | 330(164,723) | 679.5(340,1258) | **<0.001** |
| Ferritin (ng/mL) | 414.85(188.3,789) | 144.5(31.9,277.3) | 425.8(237,747.4) | 619.3(395,1056) | **<0.001** |
| Lactate dehydrogenase (U/L) | 289(188,433) | 153(121,205) | 292(224.9,411.4) | 416.5(321,484.8) | **<0.001** |
| C reactive protein (mg/L) | 18.07(5,69.45) | 3.1(1.4,9.7) | 57.15(7.57,144.67) | 23.33(14.45,89.6) | **<0.001** |

*Kruskal-Wallis Test. Median (IQR), interquartile range.

Table 3S.- Clinics and biomarkers characteristics stratified by allelic and genotype of rs2285666 population of study

|  | Mild  n=132 (45%) | P | Severe n=115 (33%) | P | Critical  n=199 (22%) | P |
| --- | --- | --- | --- | --- | --- | --- |
| Oxygen saturation % |  |  |  |  |  |  |
| C | 94(92,95) | 0.58 | 88 (78,92) | 0.85 | 82 (74,89) | **0.66** |
| T | 94(92,96) |  | 86 (74,94) |  | 83 (70,88) |  |
| CC | 94(92,95) | 0.84 | 88 (78,92) | 0.90 | 82 (73.5,88.5) | 0.28 |
| CT | 94(92,95.5) |  | 87 (83,92) |  | 85 (79,90) |  |
| TT | 94(92,96) |  | 85 (70,94) |  | 83 (69.5,88) |  |
| D-dimer (ng/mL) |  |  |  |  |  |  |
| C | 253(152.5,443) | **0.02** | 324 (165,723) | 0.81 | 663.5 (273,1217) | 0.44 |
| T | 323(217,510) |  | 358 (150,685) |  | 696.5 (374,1447) |  |
| CC | 235.5(150,386) | **0.03** | 323.5 (152.5,731.5) | 0.94 | 688.5 (238,1217) | 0.84 |
| CT | 360(214.5,555) |  | 420 (210,621) |  | 612 (402,1257) |  |
| TT | 285(217,430) |  | 356 (116,739) |  | 759.5 (374,1468) |  |
| Ferritin (ng/mL) |  |  |  |  |  |  |
| C | 144.05(34,274.8) | 0.83 | 452 (259.8,885.9) | **0.02** | 569.5 (370.4,1021.6) | 0.06 |
| T | 145.1(30.35,306.6) |  | 393.8 (206.4,630.8) |  | 637.5 (405.3,1108.5) |  |
| CC | 173.7(61,313.6) | **0.01** | 482.05(290.65,984.95) | **0.04** | 609.7 (409.7,1033) | **<0.001** |
| CT | 72.55(12.7,152.55) |  | 305.3 (121,443.8) |  | 433.3 (276.5,628.2) |  |
| TT | 167(45.05,152.55) |  | 420.4(206.4,659.4) |  | 799.8 (441.7,1301) |  |
| Lactate dehydrogenase (U/L) |  |  |  |  |  |  |
| C | 152.5(121,202.5) | 0.71 | 299 (226,428) | 0.41 | 424 (317,474.1) | 0.62 |
| T | 157(122,216) |  | 280.4 (213.3,411.4) |  | 407 (324,530) |  |
| CC | 153.5(123,207) | 0.41 | 314.31(233.5,484.5) | **0.04** | 425.1 (324.55,475.05) | 0.21 |
| CT | 147.5(63.85,185.5) |  | 257(166,282.1) |  | 381 (257,438) |  |
| TT | 163(125,222) |  | 322.8(213.3,439) |  | 407.5 (326.5,535.8) |  |
| C reactive protein (mg/L) |  |  |  |  |  |  |
| C | 2.8(1,8.3) | **0.006** | 47.5 (7.42,140) | 0.29 | 22.74 (13.15,89.6) | 0.24 |
| T | 4.95(2,13.4) |  | 69.45 (12,150.41) |  | 23.98 (16.06,90) |  |
| CC | 2.8(0.9,8.4) | 0.07 | 49.44(7.42,142.8) | 0.60 | 23.33 (14.45,118.69) | **0.01** |
| CT | 5.4(2.4,9.1) |  | 32.95 (6.68,105.06) |  | 16.3 (11.66,23.98) |  |
| TT | 5.3(2.4,16.2) |  | 69.7 (12.56,175.71) |  | 26.55 (16.61,113.19) |  |

*Kruskal-Wallis Test. Median (IQR), interquartile range.
